# Profiling Potential Wine Yeast Starters from *Criolla* Grape Varieties from Argentina

**DOI:** 10.3390/jof12050322

**Published:** 2026-04-28

**Authors:** Lucía Maribel Becerra, Carolina Torres Palazzolo, Selva Valeria Chimeno, María Cecilia Lerena, Santiago Sari, Jorge Prieto, Laura Analía Mercado, Mariana Combina

**Affiliations:** 1 Consejo Nacional de Investigaciones Científicas y Técnicas (CONICET), Buenos Aires C1425FQB, Argentina; becerra.lucia@inta.gob.ar (L.M.B.); ctorrespalazzolo@fca.uncu.edu.ar (C.T.P.); lerena.cecilia@inta.gob.ar (M.C.L.); 2 Estación Experimental Agropecuaria Mendoza, Instituto Nacional de Tecnología Agropecuaria (INTA), Luján de Cuyo 5507, Argentina; chimeno.valeria@inta.gob.ar (S.V.C.); sari.santiago@inta.gob.ar (S.S.); prieto.jorge@inta.gob.ar (J.P.); 3 Facultad de Ciencias Agrarias, Universidad Nacional de Cuyo, Chacras de Coria M5528AHB, Argentina; 4 Centro de Estudios Vitivinícolas y Agroindustriales (CEVA), Universidad Juan Agustín Maza, Guaymallén 5519, Argentina

**Keywords:** *Saccharomyces*, non-*Saccharomyces*, oenological characterisation, autochthonous yeasts, wine starters

## Abstract

*Criolla* grape varieties are native South American cultivars that represent an important reservoir of genetic and microbiological diversity. This study aimed to investigate the oenological potential of *Saccharomyces* and non-*Saccharomyces* yeasts isolated from three *criolla* grape varieties in order to support the future design of wine starters. Yeasts were isolated at different fermentation stages from four vineyards in Mendoza, Argentina. A total of 485 isolates were recovered and molecularly identified, revealing 12 species belonging to eight genera. *Saccharomyces cerevisiae*, *Hanseniaspora guilliermondii* and *Hanseniaspora uvarum* were the dominant species. Isolates were screened for H_2_S and acetic acid production, followed by physiological and enzymatic characterisation. Selected strains were further evaluated in small-scale fermentations to assess fermentative kinetics and metabolic performance. Significant variability was observed, particularly among non-*Saccharomyces* isolates, which generally exhibited lower ethanol yields and acetic acid production compared to *S. cerevisiae*. Several isolates of *H. guilliermondii* showed balanced fermentative behaviour and favourable metabolic and enzymatic profiles. Through the applied selection strategy, twelve strains emerged as promising wine starter candidates. These isolates combined a low production of undesirable metabolites, relevant enzymatic activities, and favourable fermentative performance. Overall, the results highlight the oenological potential of autochthonous yeasts as a resource for innovative winemaking strategies.

## 1. Introduction

The name “*criolla* grapes” refers to the native grape varieties (*Vitis vinifera* L.) originating from South America, resulting from hybridization through natural crossbreeding between the grapevines brought over by the Spanish. These natural crossings gave rise to new genotypes that have been cultivated for over 400 years in the vineyards of Argentina and other regions in America [1,2]. Some of these genotypes have evolved and adapted to local conditions. Therefore, it is likely that the genetic diversity found in America serves as a reservoir for new genotypes and ancient genotypes lost in Europe [3]. In line with this, the National Institute of Agricultural Technology (INTA) has a germplasm collection of grapevines in the Mendoza Experimental Station that preserves *criolla* grape varieties. Their molecular characterisation showed that the diversity of South American cultivars is greater than previously considered, where 18 varieties were original genotypes, without homology in reference databases [3]. Germplasm collections play a key role in the preservation and characterisation of genotypes that would otherwise have been lost [2].

At present, approximately 25% of the cultivated vineyard area in Argentina is planted with *criolla* grape varieties [4]. In the past, they were massively planted due to their high yield and resistance to abiotic factors, mainly to produce limited quality wines. However, changes in wine demand and consumption patterns, increasingly oriented towards lighter and more aromatic wines suited to diverse consumption occasions, call for innovation and diversification in winemaking, in which *criolla* grape varieties represent a valuable opportunity [2,5]. The use of these grape varieties is of particular interest in the context of wine market diversification and innovation, and it has been prioritised in the Argentine Wine Strategic Plan 2030 [6].

Yeasts are part of the natural microbiota of grapes and play a key role in the fermentation of musts [7]. The yeasts drive alcoholic fermentation and contribute to wine aroma complexity through the production of secondary metabolites, thereby shaping wine typicity and regional identity [7,8,9,10]. The main two groups of oenological yeasts involved are the *Saccharomyces* genus, particularly *Saccharomyces cerevisiae*, with a key role in must alcoholic fermentation, and non-*Saccharomyces* (NS) yeasts, a heterogeneous group which have recently been studied for their positive effects on wine fermentation [11].

*Saccharomyces cerevisiae* strains have been selected in different viticultural regions and are widely used as starter inoculum for alcoholic fermentation, providing reliable control over fermentation kinetics and contributing to the desired aromatic profile of the wine [7,8,12]. Increasing interest has emerged in the use of indigenous yeasts adapted to local grape varieties and winemaking conditions, reflecting the concept of microbial *terroir*, a context where NS yeasts have recently attracted considerable attention [13,14]. These NS yeasts have been highlighted for their significant contribution to the chemical composition and organoleptic properties of wine [9,11,15,16,17]. Furthermore, research on NS strains has aimed to exploit oenological advantages that are absent or less favoured in *S. cerevisiae* [11]. A key aspect is their contribution to the volatile and sensory diversity of wines, which represents oenological potential of considerable importance [17,18,19]. Different studies have previously addressed the characterisation of enzymatic activities in NS strains, reporting high enzymatic activities such as proteases, pectinases, β-glucanases, β-glucosidases and cellulases. The diversity and high enzymatic activity within different oenological NS strains prompt them as boosters to improve wine organoleptic quality in a strain-dependent manner [20,21,22]. Additionally, in the current trend of low-ethanol wines, NS strains have been proposed as an attractive strategy to reduce the ethanol content in wines, since NS have different respiro-fermentative regulatory systems that allow them to direct carbon source utilisation toward the formation of products different from ethanol. Various strains such as *Metschnikowia pulcherrima*, *Starmerella bacillaris*, *Candida californica*, and *Hanseniaspora uvarum* have been already evidenced as good candidates for limited ethanol reduction [15,16,23]. Other NS yeast species have been investigated as potential tools for winemaking including *H. uvarum*, *H. guilliermondii*, *Lachancea thermotolerans*, *M. pulcherrima*, *Schizosaccharomyces pombe* and *Torulaspora delbrueckii*, which in the past were not considered for winemaking applications. Current research has increasingly focused on their use in combination with *S. cerevisiae*, highlighting their role as starters cultures in mixed or sequential fermentations, where they can enhance wine complexity while *S. cerevisiae* ensures the completion of fermentation [8,13,14,24,25,26,27,28,29,30].

Each viticultural ecosystem represents a unique opportunity to explore yeast genetic diversity, enabling the genuine expression of distinctive wine attributes [10,31,32]. Characterising *Saccharomyces* and NS yeast populations associated with specific viticultural ecosystems is thus of interest for improving our understanding of the winemaking process and developing oenological strategies to modulate wine characteristics according to product objectives [8,12].

Currently, there are no selected autochthonous yeasts for the fermentation of Argentine *criolla* grape varieties. Some local wineries rely on spontaneous fermentations to preserve and enhance the distinctive aromatic expression of these cultivars. However, this approach involves certain risks and often results in organoleptic profiles that are difficult to reproduce. Conversely, other wineries prefer the use of commercially available starter cultures, which ensure fermentation reliability and consistency, but may lead to a loss of distinctive sensory characteristics in the resulting wines. Therefore, the selection of autochthonous yeasts that enhance the desired attributes of *criolla* wines represents a valuable technological strategy for adding value and diversifying production.

The aim of this study was to explore the oenological potential of *Saccharomyces* and NS yeasts isolated from three Argentine *criolla* grape varieties for the design of future wine autochthonous starters.

## 2. Materials and Methods

### 2.1. Vineyards

Three widely distributed *criolla* grape varieties from four vineyards were selected for this study. Moscatel Blanco, Criolla Grande, and Pedro Giménez were sampled from an ancient vineyard established in 1790 in Junín, Mendoza, Argentina (−33.11788, −68.42862), managed under a pergola training system. In addition, Criolla Grande grapes were also sampled from a vineyard located in Vistaflores, La Consulta, Mendoza, Argentina (−33.66380, −69.15137).

### 2.2. Spontaneous Fermentations

Grapes were hand-harvested; their basic composition was adequate, indicating healthy, mature grapes: Moscatel: 17.4 ± 0.2 °Brix; 4.71 ± 0.86 g/L tartaric acid; pH 3.66 ± 0.12; Pedro Gimenez: 24.4 ± 0.2 °Brix; 4.54 ± 0.21 g/L tartaric acid; pH 3.93 ± 0.5; Criolla grande from Junin: 25 ± 0.13 °Brix; 4.79 ± 0.18 g/L tartaric acid; pH 3.65 ± 0.25; and Criolla grande from Vistaflores 22.3 ± 0. 3 °Brix; 4.54 ± 0.02 g/L tartaric acid; pH 4.54 ± 0.2. Grapes were immediately destemmed and crushed before being transferred to 25 L plastic fermentation vessels. Spontaneous fermentations were conducted in duplicate without the addition of commercial yeast or sulphur dioxide. Fermentations were carried out at 28 ± 1 °C by placing the vessels in a temperature-controlled room and daily monitored by measuring tank weight loss due to CO_2_ release and changes in must density.

### 2.3. Sampling and Yeast Isolation

Samples for yeast isolation were collected at three fermentation stages: the beginning of fermentation, mid-fermentation (50% residual sugar), and the end of fermentation (residual sugar < 4 g/L). At each stage, 250 mL samples were aseptically collected in sterile glass containers. Sample aliquots were serially diluted in 0.1% (*w*/*v*) peptone water and plated onto WL Nutrient Agar (Oxoid, Basingstoke, UK) supplemented with chloramphenicol (50 µg/mL) and incubated at 28 ± 1 °C for 72 h, for yeast differentiation, enumeration, and isolation. Colonies were selected based on macroscopic morphological characteristics, and around 20 representative colonies from sample population were purified by subculturing on YPD agar (Agar 2 g/L, Yeast Extract 5 g/L, Peptone 5 g/L and Dextrose 40 g/L), and later on Lysine agar medium (Oxoid) in order to confirm *Saccharomyces*/non-*Saccharomyces* assignment. A total of 485 isolates were stored in 30% (*v*/*v*) glycerol at −20 °C until identification.

### 2.4. Yeast Identification

Frozen cultures of all yeast isolates were streaked onto YPD agar plates and incubated for 48 h. A single isolated colony was picked to inoculate 5 mL of YPD medium and incubated overnight at 28 ± 1 °C. The samples were centrifuged and washed with sterile Milli-Q water to prepare them for DNA extractions using the method previously described [33]. A preliminary identification of representative isolates was performed by RFLP of the amplified 5.8S-ITS region [34,35]. For this purpose, the 5.8S-ITS fragments were amplified using primers ITS1 (5′-TCCGTAGGTGAACCTGCGG-3′) and ITS4 (5′-TCCTCCGCTTATTGATATGC-3′) following the PCR programme: 5 min at 95 ˚C, 40 cycles of 94 °C for 1 min, 55.5 °C for 2 min, and 72 °C for 2 min, and final elongation at 72 °C for 10 min. Amplification and the size of amplicons were evaluated by migration/separation in 1.4% agarose gels. PCR products were digested with the restriction endonucleases *Cfo*I, *Hae*III and *Hinf*I (New England Biolabs Co., Ltd., Ipswich, MA, USA), endonuclease *Dde*I (New England Biolabs Co., Ltd., Ipswich, MA, USA) was additionally used for apiculate yeast identification. The restriction products were migrated on 3% agarose gels. Digestion profiles were interpreted and analysed with yeast-ID database [36]. Subsequently, representatives isolates (n = 96) from each restriction profile were confirmed by Sanger sequencing (Macrogen Co., Ltd., Seoul, Republic of Korea). Species identity was determined using the BLAST (version 2.17.0) tool in NCBI (https://blast.ncbi.nlm.nih.gov/Blast.cgi accessed on 20 October 2025). A minimum of 99% of sequence identity with the type strain of a yeast species was required for species identification [37,38]. Sequencing of the D1/D2 domain of the 26S ribosomal RNA gene was performed to confirm species identity for isolates that had previously shown uncertain or ambiguous identification (n = 40). A sequence identity of ≥99% with the type strain was again considered the criterion for species assignment using BLAST analysis in the NCBI database.

### 2.5. Oenological Characterisation of Yeast Isolates

The production of undesirable compounds, such as hydrogen sulphide (H_2_S) and acetic acid (AA), was evaluated as a primary criterion to exclude isolates with low oenological potential. The ability of yeast strains to produce H_2_S was assessed on BIGGY agar plates (Oxoid) a commercially available bismuth-containing medium. Yeast isolates were streaked for single colonies onto BIGGY agar and incubated at 28 ± 1 °C for 48–72 h. Hydrogen sulphide production was estimated based on the degree of colony blackening, which provides a visual indicator of the genetically determined maximal sulphite reductase activity and the potential of each strain to produce H_2_S under permissive conditions [39,40]. Colony colour was evaluated using a five-level scale ranging from 0 (black, high H_2_S production) to 4 (uncoloured, low or null H_2_S production). Accordingly, the coding scheme assigned lower numerical values to higher H_2_S production.

Acetic acid production was assessed on Yeast Glucose Agar supplemented with calcium carbonate (YGA–CaCO_3_: Agar 2 g/L, Yeast Extract 5 g/L, CaCO_3_ 10 g/L and Dextrose 20 g/L), by spot inoculation of 5 μL of yeast cultures in the exponential growth phase. Acetic acid production results in the dissolution of calcium carbonate, generating a clear halo around the colonies [41]. Halo diameter was used as a semi-quantitative indicator, and strains were classified according to a four-level scale ranging from 0 to 3. The scale was coded so that 0 corresponded to the highest acetic acid production (least desirable behaviour), whereas 3 corresponded to the lowest production (most desirable behaviour). Only strains that simultaneously exhibited H_2_S and AA production scores ≥ 2 were selected for further analyses.

Yeast isolates pre-selected were evaluated for their ability to grow under oenologically relevant stress conditions, including increasing ethanol and sulphur dioxide concentrations, osmotic stress tolerance, growth at low and high temperatures (15 and 35 °C), and acidic pH. The activities of enzymes of oenological interest, including β-glucosidase, pectinase, and protease, were also evaluated. Each assay was carried out in triplicate.

All yeast isolates were previously grown in YPD broth at 28 ± 1 °C for 48 h. Subsequently, each isolate was inoculated onto the specific solid media used for each determination by replica plating at a concentration of 1 × 10^6^ cells/mL. YPD agar plates without supplementation were also inoculated and used as growth controls.

#### 2.5.1. Ethanol and SO_2_ Tolerance

Each yeast isolate was inoculated onto YPD agar adjusted to pH 3.0 and supplemented with potassium metabisulphite. For NS yeast isolates, concentrations of 100, 200, and 300 ppm were tested, whereas for *Saccharomyces* isolates, 200, 300, and 500 ppm were evaluated. In parallel, ethanol tolerance was assessed on YPD agar supplemented with increasing ethanol (EtOH) concentrations. NS yeast isolates were tested at 10%, 11%, 12%, and 13% (*v*/*v*), while *Saccharomyces* isolates were evaluated at 12%, 13%, 14%, and 15% (*v*/*v*). Plates were incubated at 28 ± 1 °C and growth was evaluated after 48 h.

#### 2.5.2. Osmotic Stress Tolerance

YPD agar supplemented with high glucose concentrations was used to assess osmotic stress tolerance. Glucose was added at 250 g/L for NS yeast isolates and 300 g/L for *Saccharomyces* isolates. Plates were incubated at 28 ± 1 °C and growth was evaluated after 48 h.

#### 2.5.3. Growth at Low and High Temperatures

Strains were inoculated onto YPD agar and incubated at 15 ± 1 °C and 35 ± 1 °C to assess growth at low and high temperatures. Yeast growth was evaluated after 48–72 h of incubation.

#### 2.5.4. Growth at Acidic pH

YPD agar adjusted to pH 3.5 with 0.1 M HCl was used to assess growth under acidic conditions. Plates were incubated at 28 ± 1 °C for 24 h.

#### 2.5.5. Evaluation of Enzymatic Activities

##### β-Glucosidase Activity

This enzymatic activity was evaluated qualitatively using esculin as substrate, following methodology previously described [42]. Plates were incubated at 28 ± 1 °C for 48–72 h. β-Glucosidase activity was considered positive when a dark brown to black halo appeared around the colonies, resulting from the formation of an esculetin–iron complex.

##### Pectinase Activity

This assay was performed according to Fernandes-Salomão et al. [43] and Merín et al. [44] on agar plates. Plates were incubated at 28 ± 1 °C for 72 h. After incubation, plates were flooded with 30 mL of Lugol’s iodine solution, and pectinase activity was detected by the presence of clear halo surrounding the colony, indicating pectin degradation.

##### Protease Activity

The methodology previously described by Comitini et al. [45] was followed. Plates were incubated at 28 ± 1 °C for 72 h. Protease activity was indicated by the presence of a translucent halo surrounding the colony, corresponding to casein degradation.

### 2.6. Small-Scale Wine Fermentation

Yeast isolates selected based on the oenological and enzymatic characterisation were further evaluated for their fermentative performance in small-scale wine fermentations. Strain selection was based not only on oenological characteristics but also on origin of isolation, fermentation stage, and species identity. Yeast inocula were prepared starting from cryopreserved stocks. Each strain was streaked onto YPD agar plates and incubated at 28 ± 1 °C for 48 h. Fresh biomass was then transferred into test tubes containing 5 mL of YPD broth adjusted to pH 4.5 with tartaric acid solution (15% *w*/*v*) and incubated at 28–30 °C under agitation for 3 h. Subsequently, the entire culture was transferred to sterile flasks containing 40 mL of medium composed of grape must (8 °Brix), supplemented with yeast extract 2 g/L, peptone 4 g/L, glucose 40 g/L and adjusted to pH 4.5 with tartaric acid. Cultures were incubated overnight at 28–30 °C with agitation (160 rpm).

Small-scale fermentations were carried out using pure yeast cultures inoculated at an initial population of 2 × 10^6^ cells/mL on Erlenmeyer flasks containing 300 mL of grape must adjusted to 25 Brix and pH 3.5, with the addition of diammonium phosphate (NH_4_)_2_HPO_4_ at a final concentration of 200 mg/L. Flasks were aseptically sealed with Müller valves. All fermentations were performed in triplicate and incubated under static conditions at 28 ± 1 °C.

Fermentation kinetics were monitored daily by measuring weight loss due to CO_2_ release until constant weight was reached. In parallel, qualitative observations such as foam formation, sedimentation, and flocculation were recorded. At the end of fermentation, physicochemical parameters including ethanol (% *v*/*v*), total sugars (g/L), and acetic acid (g/L) were determined using OENO FOSS wine scan (Hilleroed, Denmark). Based on the obtained data, the following fermentation performance parameters were calculated: acetic acid production (g/L); sugar uptake (expressed as the percentage of initial sugars consumed); fermentative yield (expressed as the ratio between sugar consumed and ethanol produced, % *v*/*v*); fermentative efficiency (%, expressed as the ratio of ethanol produced and the theoretical ethanol yield based on the initial must sugar concentration). The theoretical ethanol value was calculated considering that 16.83 g/L of sugar is required to produce 1% (*v*/*v*) ethanol [7]. Additionally, fermentative kinetic parameters were calculated by fitting the fermentation curves to a non-linear regression model based on the Gompertz equation, according to the following expression:

(1) $$y=A\times exp\{-exp[(\left(\mu_{max}\times e/A\right)\times \left(\lambda -t\right))+1]\}$$

The parameters of the fitted growth model can be biologically interpreted as the lag phase (λ and the maximum specific growth rate (µ_max_). The parameters were further reassigned to fermentation kinetics, where λ represents the lag time (i.e., the time required for fermentation to start vigorously) and µ_max_ corresponds to the maximum fermentation rate, expressed as the highest percentage of weight loss per unit time.

### 2.7. Statistical Analysis

For H_2_S and AA production, yeast isolates were classified into ordinal categories according to their production levels, as previously described. To explore the association between yeast species and the categorical levels of H_2_S and acetic acid (AA) production, a Correspondence Analysis (CA) was performed. This multivariate technique allowed for a graphical representation of the relationships between species and undesirable metabolite production categories. Furthermore, a classification tree based on the deviance criterion was constructed using species as the response variable and H_2_S and AA categorical scores as splitting variables. This analysis was intended as a supervised multivariate selection procedure based on ordinal phenotypic scores, aimed at identifying isolates with desirable oenological traits. Data processing and graphical analyses were performed using Microsoft Excel, version 2604 (One Microsoft Way, Redmond, WA, USA) and InfoStat software version 2020i (Universidad Nacional de Córdoba, Córdoba, Argentina) [46].

In the second stage of the screening process, preselected isolates were further characterised under stress growth conditions and for enzymatic activities. The resulting data were used to construct a classification tree based on the deviance criterion, using species as the response variable and stress tolerance responses together with enzymatic activity profiles as splitting variables. This analysis enabled the partitioning of isolates according to their combined phenotypic attributes, facilitating the identification of groups with similar oenological performance and supporting the final selection of strains for fermentative trials.

Kinetic fermentative parameters, namely maximum fermentation rate and lag phase duration, were analysed using R software version 4.5.3 (R Foundation for Statistical Computing, Vienna, Austria). Kinetic parameters, including maximum fermentation rate and lag phase duration, were estimated by fitting fermentation curves to the non-linear model. The goodness of fit of each model was evaluated using the coefficient of determination (R^2^). Subsequently, the estimated fermentative parameters were statistically compared among yeast strains by analysis of variance (ANOVA) using InfoStat software [46]. Differences were considered statistically significant at *p* < 0.05. Then, principal component analysis (PCA) was performed to explore the multivariate relationships among yeast isolates based on fermentative kinetic and metabolic parameters obtained from small-scale fermentations. The analysis included lag phase duration (λ), maximum fermentation rate, sugar consumption, fermentation efficiency, fermentative yield, and acetic acid production. Prior to PCA, variables were standardised to avoid scale effects. The analysis was conducted using InfoStat software, and the first two principal components were used to visualise the distribution of isolates and the contribution of the evaluated variables through a biplot representation.

Finally, a multi-response desirability function approach was applied to support the selection of the most promising yeast isolates for wine fermentation. Data obtained from small-scale fermentation assays were analysed using Design-Expert software version 8.0.6 (Stat-Ease Inc., Minneapolis, MN, USA), considering the yeast isolate as a categorical factor in a full factorial design. Two selection scenarios were defined according to different technological objectives. The first scenario prioritised fast growth, short lag phase, complete sugar consumption, low ethanol and acetic acid production, and high fermentative yield, aiming to favour the formation of metabolites other than ethanol. The second scenario prioritised fast growth, short lag phase, complete sugar consumption, low acetic acid production, and maximum ethanol production, favouring efficient sugar-to-ethanol conversion. In both cases, isolates were ranked according to their overall desirability, allowing for the identification of strains with differentiated fermentative profiles.

## 3. Results

### 3.1. Yeast Isolation and Identification

A total of 485 yeasts were recovered from samples collected at different stages of spontaneous fermentations of four *criolla* grapes samples, conducted in duplicate. The applied isolation strategy resulted in a broad representation of yeast diversity, reflecting a potential wide range of phenotypic traits. Of the total isolates, 307 were classified as *Saccharomyces*, while 178 were assigned to the non-*Saccharomyces* (NS) group based on their ability to grow on lysine differential medium.

**Figure 1:**
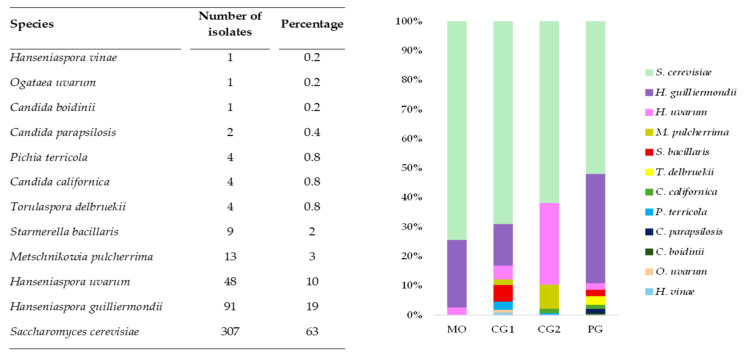
Frequency of yeast species isolated and their distribution from different *criolla* grape varieties. MO: Moscatel blanco; CG1: Criolla Grande 1; CG2: Criolla Grande 2; PG: Pedro Gimenez.

All yeast isolates were subjected to molecular identification. Initially, 5.8S-ITS RFLP analysis was performed. PCR products were digested with restriction endonucleases, generating a total of 12 distinct restriction profiles, which allowed the isolates to be grouped accordingly. Complete RFLP profiles and the distribution of isolates among restriction profiles are provided in the Supplementary Material (Table S1). Representative isolates from each RFLP profile were identified by sequencing the 5.8S-ITS region and, when necessary, confirmed by sequencing the D1/D2 domain of the 26S rRNA gene. Overall, 8 genera and 12 yeast species were identified. However, three species predominated, accounting for approximately 92% of the total isolates. The most frequently isolated species was *S. cerevisiae* (63%), followed by *H. guilliermondii* (19%) and *H. uvarum* (10%). These species were detected in all grape varieties analysed (Figure 1). The remaining 8% of the isolates were distributed among *M. pulcherrima*, *S. bacillaris*, *T. delbrueckii*, *C. californica*, *Pichia terricola*, *Candida parapsilosis*, *Candida boidinii*, *Ogataea uvarum and H. vinae.*

All identified yeasts were incorporated into the INTA Mendoza Microorganisms Collection (CoMIM), housed at INTA Mendoza and integrated within the INTA National Microbial Genetic Resources Network, in line with the strategic objective of preserving native genetic resources.

### 3.2. Oenological Traits Characterisation

#### H_2_S and Acetic Acid Production

**Figure 2:**
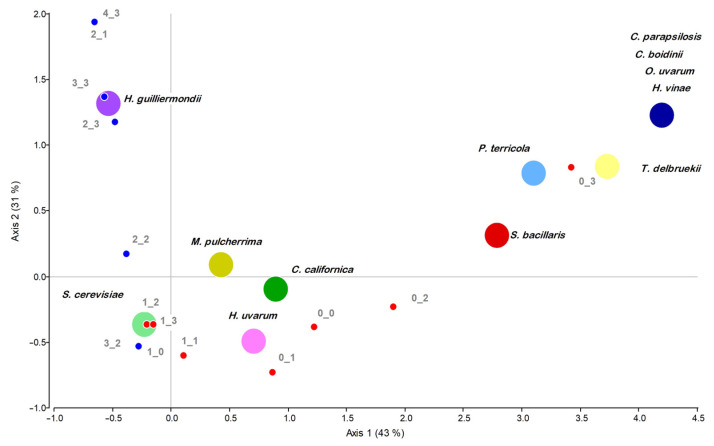
Correspondence analysis biplot based on combined H_2_S and AA production scores. Circles of different colours represent different species. Small dots represent the combined H_2_S_AA score categories. Blue dots indicate the score combinations selected for further characterisation (H_2_S_AA scores ≥ 2), while red dots indicate excluded combinations.

The initial screening was based on the production of undesirable compounds, specifically H_2_S and acetic acid (AA). The biplot generated by Correspondence Analysis (Figure 2) shows that along the first axis (43% inertia), *S. cerevisiae* and *H. guilliermondii* isolates are positioned in association with the lowest production levels of both undesirable metabolites. Along the second axis (31% inertia), *H. guilliermondi* appears more closely associated with the low-production categories, reflecting a higher proportion of isolates with reduced H_2_S and acetic acid production compared to *S. cerevisiae.*

**Figure 3:**
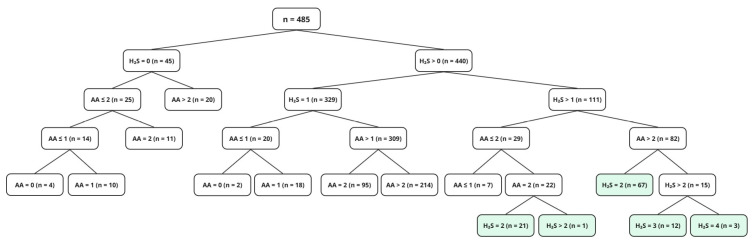
A classification tree constructed using species as the response variable and H_2_S and AA categorical scores as splitting variables (deviance criterion). Terminal nodes represent combined production profiles and show the number of isolates (n) within each profile. Light green boxes indicate the selected isolates.

A classification tree was constructed to evaluate these parameters (Figure 3). The resulting terminal nodes represented distinct combinations of undesirable metabolite production levels and showed the distribution of isolates from each species within each profile. H_2_S production emerged as the main factor determining strain exclusion. Consequently, 100% of the isolates of *H. vinae*, *O. uvarum*, *P. terricola*, *Candida* spp., and *S. bacillaris*, 96% of *H. uvarum*, 88% of *S. cerevisiae* and 85% of *M. pulcherrima* isolates were excluded due to low scores (0–1), corresponding to high H_2_S production. In contrast, AA production scores showed that most isolates from both groups clustered in the highest score category (score 3), corresponding to low acetic acid production, with only a small proportion of strains exhibiting high production levels. Among the isolates exhibiting null to low H_2_S production, 94% also displayed null to low acetic acid production (scores 2–3) and were therefore selected for further characterisation (light green boxes in Figure 3).

These results guided the pre-selection of 104 isolates for the next stage, which included a more detailed oenological characterisation. The selected isolates belonged to four species: *H. uvarum* (2), *M. pulcherrima* (2), *S. cerevisiae* (35), and *H. guilliermondii* (65).

### 3.3. Growth Under Oenological Conditions and Enzymatic Activities

The ability of the 104 pre-selected yeast isolates to grow under oenologically relevant stress conditions, including increasing ethanol and sulphur dioxide concentrations, osmotic stress, and growth at low and high temperatures as well as at acidic pH, was evaluated. In addition, the activity of enzymes of oenological interest, namely β-glucosidase, pectinase, and protease were assessed. Plate-based screening of β-glucosidase can be performed using different chromogenic substrates that produce brown to black pigments in the presence of the enzyme [47]. In the present study, an esculin-based assay was employed as a rapid screening method to classify the yeast isolates, taking into account the inherent limitations of this approach.

For the analysis of phenotypic profiles, a binary matrix was constructed in which the following were considered positive oenological traits (coded as 1): tolerance to the maximum concentrations of ethanol and sulphur dioxide evaluated; growth under osmotic stress conditions, at low and high temperatures, and at acidic pH; and the presence of enzymatic activities of oenological interest. The absence of these traits was coded as 0. A total of nine traits were evaluated, generating 11 statistically distinct phenotypic profiles (Table S2). Regarding *S. cerevisiae*, 74% of the isolates (26/35) exhibited a phenotypic profile accumulating most positive oenological traits, except for β-glucosidase and protease activities. The remaining isolates displayed higher enzymatic diversity: 6% (2/35) showed both β-glucosidase and protease activities, 14% (5/35) exhibited only β-glucosidase activity, and 6% (2/35) showed only protease activity. In the case of *H. guilliermondii*, the most abundant group evaluated in this stage (65 isolates), phenotypic profiles were more heterogeneous, resulting in nine distinct trait combinations. The most frequent profile, representing 42% of the isolates (27/65), accumulated seven positive oenological traits and was characterised by the presence of β-glucosidase activity, but ethanol sensitivity, and absence of protease activity. The second most frequent profile (23%, 15/65) accumulated all desired oenological traits. Additional clusters (each representing 9% of the isolates) either lacked ethanol tolerance or lacked protease and pectinase enzymatic activities. Overall, the majority of *H. guilliermondii* isolates exhibited β-glucosidase activity, while showing variability in ethanol tolerance, pectinase and protease activities. Regarding minority species, both *M. pulcherrima* isolates shared an identical phenotypic profile characterised by proteolytic and pectinolytic activities and broad physiological tolerance, while lacking β-glucosidase activity. Similarly, the two *H. uvarum* isolates exhibited all desired traits except protease activity, differing from each other solely by the presence or absence of pectinase activity.

**Figure 4:**
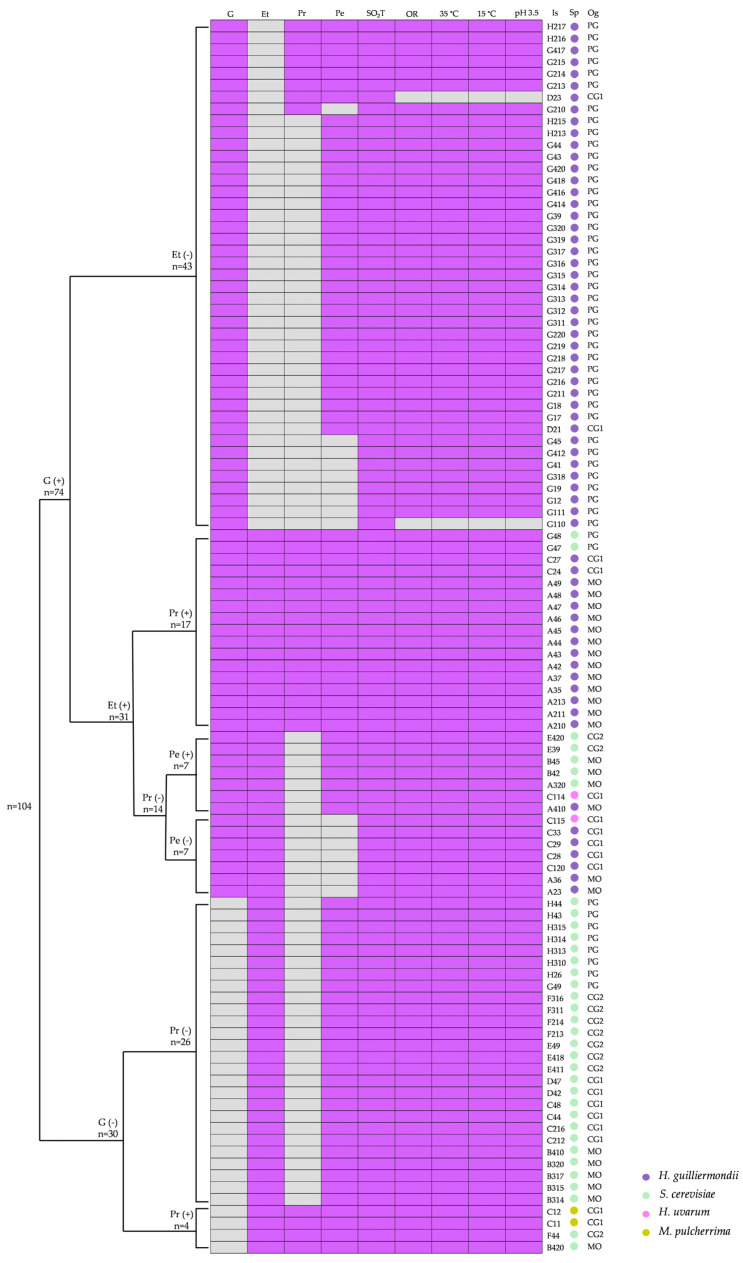
A heatmap of yeast phenotypic profiles ordered according to the terminal nodes of a supervised classification tree constructed using nine oenological traits. Binary data (presence/absence) were used to generate the heatmap, where coloured cells indicate positive traits and grey cells indicate negative traits. Coloured dots on the right indicate species (Sp) affiliation. Column abbreviations are defined below: G, β-glucosidase activity; Et, ethanol tolerance; Pr, protease activity; Pe, pectinase activity; SO_2_T, SO_2_ tolerance; OR, osmotic resistance; 35 °C, growth at 35 °C; 15 °C, growth at 15 °C; pH 3.5, growth at pH 3.5; Is, isolate; and OG, origin. MO: Moscatel blanco; CG1: Criolla Grande 1; CG2: Criolla Grande 2; PG: Pedro Gimenez.

To integrate the evaluated oenological traits, a classification tree was constructed using species as the response variable and phenotypic attributes as splitting variables (Figure 4). The analysis revealed that β-glucosidase activity was the primary splitting variable at the first node, constituting the most discriminant trait among the evaluated profiles. The β-glucosidase-negative group was composed of 80% of the *S. cerevisiae* isolates (28/35) and included both *M. pulcherrima* isolates. Within this cluster, the second discriminant trait was protease activity. In contrast, within the β-glucosidase-positive group, ethanol tolerance represented the second discriminant factor. All ethanol-sensitive isolates within this group belonged to *H. guilliermondii*, accounting for 66% (43/65) of the isolates of this species. Among ethanol-tolerant isolates, the next classification level was determined by protease activity. The protease-positive cluster (17 isolates) consisted predominantly of *H. guilliermondii* isolates (15/17) along with two *S. cerevisiae* isolates. Notably, this group of 17 isolates accumulated 100% of the desired oenological traits. Finally, the protease-negative isolates were further separated based on the presence or absence of pectinase activity. The pectinase-positive cluster was mainly composed of *S. cerevisiae* isolates, together with one *H. guilliermondii* and one *H. uvarum* isolate, whereas the pectinase-negative cluster included *H. guilliermondii* isolates and a single *H. uvarum* strain.

This oenological characterisation allowed for the selection of the most promising isolates for further evaluation in small-scale fermentations. Sixty-six strains were selected based on the most favourable profiles according to the evaluated oenological traits; while preserving species diversity, phenotypic variability identified in the classification tree analysis, and the different grape varietal origins of the isolates. According to these criteria, isolates exhibiting β-glucosidase activity were prioritised for the fermentation assay. *M. pulcherrima* isolates constituted an exception to this criterion. Although the two isolates of this species did not display β-glucosidase activity, they were positive for all other evaluated traits and were therefore included in the subsequent stage (C12 and C13). For the remaining species, a representative subset of isolates was selected according to both species affiliation and phenotypic profiles defined in the classification tree analysis. Thus, the selected strains constitute a representative sample of the main technological groups identified, while the remaining isolates were retained as material of interest for future studies. In the case of *S. cerevisiae*, all isolates displaying positive β-glucosidase activity were included. Within this group (7 isolates), two enzymatic sub-profiles were distinguished: two strains exhibiting additional pectinolytic and proteolytic activities (G47 and G48), and five strains displaying β-glucosidase and pectinolytic activities but lacking protease activity (A320, B42, B45, E39 and E420). Additionally, six isolates of *H. guilliermondii* were selected. Among them, two isolates exhibited all the desirable technological traits evaluated (C24 and C27). One isolate displayed the three enzymatic activities assessed but was sensitive to ethanol (G213). Two other isolates showed β-glucosidase and pectinolytic activities and were also ethanol-sensitive (D21 and G217). Finally, one isolate exhibited only β-glucosidase activity but demonstrated ethanol tolerance (A36). It is important to note that ethanol sensitivity was not considered an exclusion criterion for NS yeasts. Finally, one isolate of *H. uvarum* exhibiting β-glucosidase and pectinase activities, but lacking protease activity, was included (C114).

### 3.4. Small-Scale Wine Fermentation

**Figure 5:**
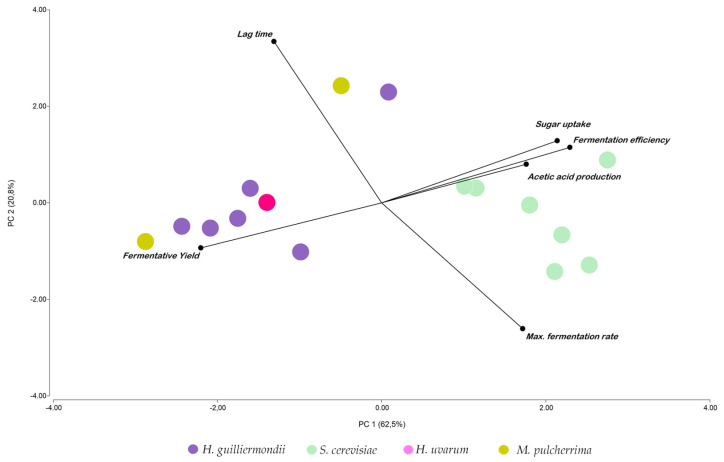
Principal component analysis (PCA) of selected yeast isolates based on fermentative kinetic and metabolic parameters obtained from small-scale fermentations. The biplot shows the distribution of isolates according to the first two principal components (PC1 and PC2), which explain 62.5% and 20.8% of the total variance, respectively. Vectors represent the contribution and direction of the evaluated variables, including lag time (λ), maximum fermentation rate, sugar uptake, fermentation efficiency, fermentative yield, and acetic acid production. Isolates are coloured according to their species affiliation.

Sixteen strains were evaluated for fermentation kinetics, sugar uptake, fermentative yield, fermentative efficiency, and acetic acid formation in grape must small-scale fermentations. The results obtained allowed for a comparative assessment of their fermentative capacity and overall performance. Initially, PCA was conducted to explore the distribution of selected isolates based on fermentative and metabolic parameters (Figure 5). The PCA revealed a tendency for isolates to cluster according to species affiliation, indicating that species-related variability contributed substantially to the overall variance. *S. cerevisiae* isolates were positioned in the direction of the vectors associated with maximum fermentation rate, fermentation efficiency, sugar uptake, and acetic acid production, suggesting comparatively higher values for these parameters. In contrast, *H. guilliermondii* and *H. uvarum* isolates tended to align with the fermentative yield vector. Two isolates, *M. pulcherrima* C11 and *H. guilliermondii* G213, were clearly separated from the main clusters and oriented towards the lag time (λ) vector, consistent with longer lag phases and comparatively lower fermentation rates. A discriminant analysis further supported the separation pattern observed in the PCA (Figure S1).

**Figure 6:**
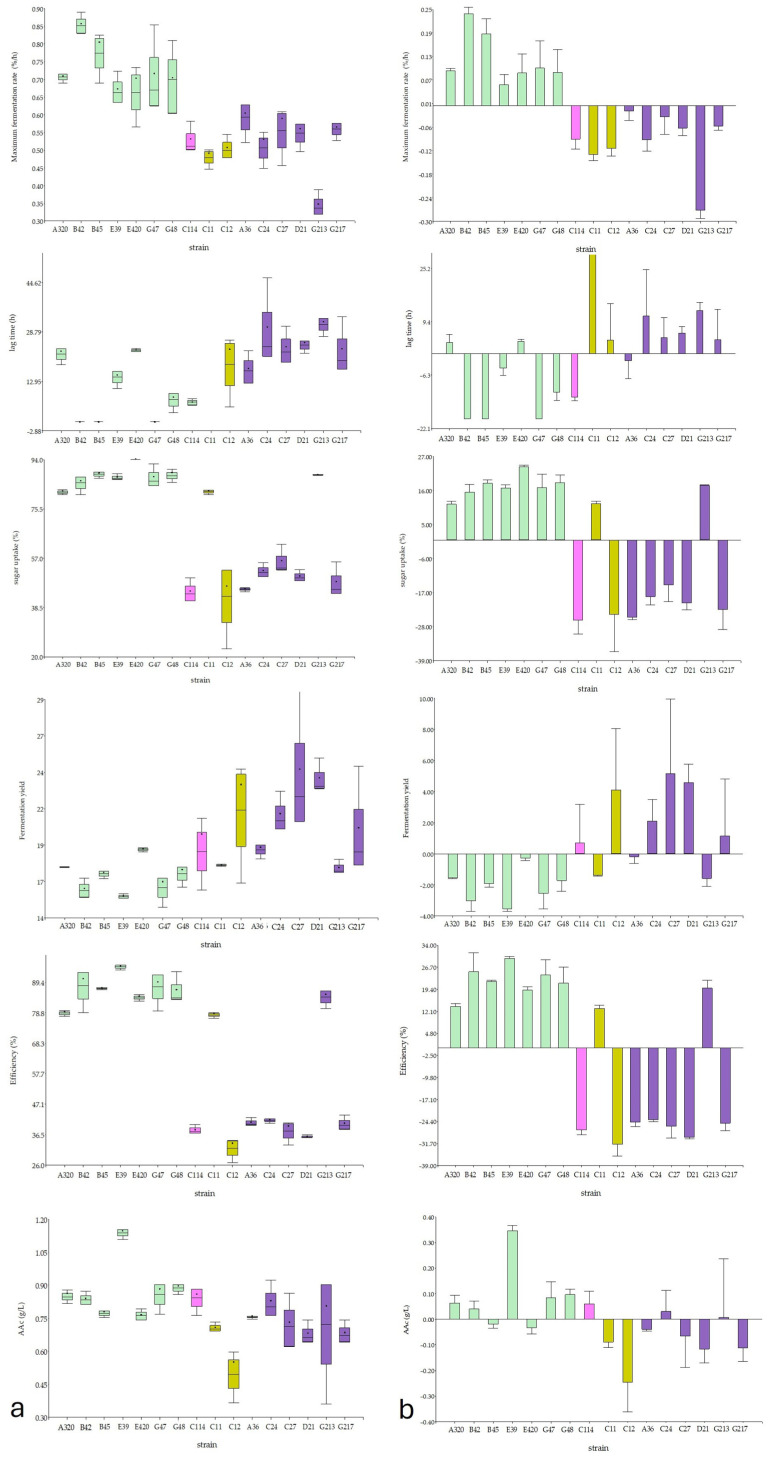
(**a**) A box plot and (**b**) mean-centred bar charts of fermentation parameters: lag time (h required to initiate fermentation), maximum fermentative rate (%/h, maximum percentage of weight loss per hour due to CO_2_ release.), sugar uptake (% of glucose consumed), fermentative yield (g of glucose consumed to produce 1% ethanol), efficiency (% ethanol produced/ethanol potentially produced) and AA (g/L of acetic acid produced). Bars of different colours represent different species.

The variability in fermentative kinetic and metabolic parameters among the selected isolates was analysed using a dual approach, combining box-plot distributions to assess population-level trends and mean-centred bar charts to visualise individual strain deviations from the global mean (Figure 6). Statistical differences were observed among strains for all evaluated parameters (ANOVA, *p* < 0.05; Table S3), highlighting a distinction between isolates regarding each fermentation parameters.

As shown by the box plots (Figure 6a), *S. cerevisiae* isolates consistently exhibited higher fermentation rates and shorter lag phases (λ) compared to most NS strains. This trend was confirmed by the mean-centred analysis (Figure 6b), where all *S. cerevisiae* isolates displayed positive deviations for fermentation rate, indicating values above the population average. In particular, *S. cerevisiae* B42 exhibited the highest positive deviation (0.25%/h) and the shortest lag phase, identifying it as the fastest-fermenting strain in the assay. Conversely, most NS isolates showed negative deviations for fermentation rate. Notably, *H. guilliermondii* G213 exhibited the largest negative deviation, corresponding to the slowest strain. Although NS strains generally displayed longer adaptation times, isolate A36 (*H. guilliermondii*) constituted an exception, showing a short lag phase comparable to that of *S. cerevisiae* strains.

An inverse relationship was observed between fermentation efficiency and fermentative yield. *S. cerevisiae* strains formed a homogeneous group characterised by high fermentation efficiencies (>88%) and low fermentative yield ratios (approximately 16–17), reflecting an efficient conversion of sugars into ethanol. In contrast, NS isolates generally exhibited lower sugar uptake and fermentation efficiencies, together with higher fermentative yield values, suggesting a redirection of carbon flux towards alternative metabolites or biomass rather than ethanol. Nevertheless, deviations from this general pattern were observed. Isolate *H. guilliermondii* G217 displayed a “*Saccharomyces*-like” profile, with higher efficiency than most conspecific isolates (Figure 6).

Regarding acetic acid production, *S. cerevisiae* strains generally produced higher levels than NS isolates in this fermentation matrix. The mean-centred analysis identified strain E39 (*S. cerevisiae*) as a clear outlier, showing the highest positive deviation for acetic acid production, whereas both *M. pulcherrima*, particularly C12, and most *H. guilliermondii* isolates showed negative deviations corresponding to lower acetic acid levels (Figure 6).

To integrate these multiple fermentation traits into a single comparative framework and support strain selection, a desirability function analysis was applied (Table S4). This approach combines multiple response variables with different technological objectives into a single overall desirability score. Based on the desirability ranking obtained (Table 1), the six highest-ranked isolates were selected under two contrasting scenarios. The first scenario, prioritising rapid growth, low ethanol and acetic acid production, and high fermentative yield, was dominated by NS yeasts, with *H. guilliermondii* C27, *M. pulcherrima* C12, *H. guilliermondii* D21 and *H. guilliermondii* G217 ranking among the top candidates. These isolates exhibited a well-balanced profile, combining favourable fermentation kinetics with low ethanol and acetic acid production, making it a promising candidate for enhancing wine complexity. Notably, despite this scenario being oriented toward metabolic diversification rather than ethanol production, two *S. cerevisiae* isolates (E420 and B45) were ranked among the most promising strains, exhibiting potential to produce wines with reduced ethanol content and low volatile acidity, despite their comparatively moderate fermentation kinetics. In contrast, the second scenario, focused on maximum ethanol production while maintaining fast growth, complete sugar consumption and low acetic acid formation, was clearly dominated by *S. cerevisiae* isolates, with B45, B42, G47, E420, G48 and A320 exhibiting the highest desirability values. Among *S. cerevisiae* strains, B42 and B45 emerged as the most suitable candidates for rapid fermentation in scenarios requiring high ethanol production due to their fast kinetics and high efficiency.

Overall, these results highlight a clear differentiation in isolate performance depending on the technological goals, reinforcing the relevance of scenario-based approaches for targeted yeast selection.

## 4. Discussion

This study contributes to a broader project focused on the valorisation and conservation of *criolla* grape varieties through an integrated framework combining agronomic, oenological, and microbiological approaches. In this context, the characterisation of the microbiota associated with these grape varieties is strategically important for wine differentiation and value addition. Understanding this microbial diversity may provide a scientific basis for enhancing regional typicity and strengthening the identity of wines produced from native cultivars [8].

For many years, the study and characterisation of oenological yeasts have been primarily focused on *S. cerevisiae*. However, NS yeasts have recently attracted increasing interest in both the scientific community and the wine industry. The use of NS yeasts in oenology has become increasingly widespread due to their ability to enhance wine quality through modifications of key physicochemical parameters. Historically, many NS species were regarded as spoilage yeasts in the wine industry [48]. However, advances in research have shifted this perception, and current approaches aim to exploit their metabolic potential to improve wine complexity rather than viewing them merely as contaminants. The intraspecific diversity of *S. cerevisiae* has been extensively explored and commercially exploited; similarly, the intraspecific diversity within key NS species offers significant opportunities to enhance and diversify wines according to contemporary consumer demand [13].

Yeasts present in four musts obtained from three *criolla* grape varieties grown in Mendoza, Argentina, were isolated, identified, and characterised. Isolation was performed during spontaneous fermentations, which enabled the recovery of abundant NS yeasts as well as diverse *S. cerevisiae* strains. The yeast species detected were consistent with those commonly reported on ripe grapes and fermentation [7]. The most frequently isolated NS species was *H. guilliermondii.* This species has not been previously detected on Malbec grapes from the same region using plate isolation methodology for yeast evaluation [49,50]. However, this species was recently reported in high abundance during spontaneous fermentations in Malbec from Mendoza using high-throughput sequencing approaches [32].

To identify suitable yeast strains for use as wine starters, an initial screening was conducted to exclude isolates exhibiting undesirable traits, such as high hydrogen sulphide (H_2_S) and acetic acid production, resulting in the retention of 21% of the isolates.

A high proportion of *S. cerevisiae* isolates exhibiting high H_2_S production scores was observed in this study, with 84% of the isolates belonging to this species excluded from the subsequent selection stage. In contrast, the capacity for H_2_S production among NS isolates was more evenly distributed across the different production categories. Specifically, 24% of NS isolates exhibited maximum H_2_S production (score 1), whereas 30% and 38% displayed intermediate and low production levels, respectively. This behaviour has been linked to strain-dependent variation in sulphur metabolism and sulphite reductase activity, which are often more evident in *S. cerevisiae* under permissive growth conditions. In contrast, many NS species have been described as low or moderate H_2_S producers, making them attractive candidates for mixed or sequential fermentations aimed at reducing sulphur-related off-flavours in wine. It has been reported that both *S. cerevisiae* and NS species differ significantly in the levels of hydrogen sulphide produced under identical physiological conditions, with variation occurring at both species and strain levels [40,45,51,52]. Regarding acetic acid production, most of the isolates were classified as low producers. Specifically, 94% of the *S. cerevisiae* isolates and 86% of the NS isolates were categorised as low or null acetic acid producers. These results agree with previous surveys focused on the characterisation and selection of wine yeasts [41,53,54].

From a practical standpoint, screening based on undesirable metabolite production efficiently reduced the number of isolates subjected to further characterisation, enabling subsequent analyses to concentrate on strains with a lower technological risk linked to sulphur and acetic acid production.

Regarding the traits of oenological interest evaluated in the 104 yeast isolates obtained from *criolla* grapes, several key enzymatic activities were identified. As previously described, the initial division of isolates was based on β-glucosidase activity, which allowed for the differentiation of two distinct clusters. As previously stated, the plate-based screening of β-glucosidase activity employed esculin as the substrate; therefore, strains showing no activity towards esculin were considered “β-glucosidase-negative”. This approach was deemed sufficient for the initial screening of the large number of strains evaluated. The β-glucosidase-negative group (30 isolates) was composed of 80% of the *S. cerevisiae* isolates (28 isolates) and the two *M. pulcherrima* isolates. In contrast, the β-glucosidase-positive group (74 isolates) included all *H. guilliermondii* isolates, the two *H. uvarum* isolates, and the remaining *S. cerevisiae* strains. β-glucosidase plays an important role in winemaking due to its impact on the release of aromatic compounds, thereby influencing wine quality [55]. Its hydrolytic activity enables the liberation of free terpenes, phenylpropenes, and specific aliphatic esters during fermentation, and may also modify the phenolic composition of wine [56]. Given the oenological interest in strains exhibiting this enzymatic activity, considerable research efforts have focused on screening yeasts with elevated β-glucosidase activity. In general, high β-glucosidase activity is considered uncommon among indigenous *S. cerevisiae* strains [53,55,57], however 20% of *S. cerevisiae* strains evaluated in the present study exhibited detectable β-glucosidase activity. In contrast, this trait was more prevalent among NS isolates, particularly within *Hanseniaspora* species, in agreement with previous reports [11,55]. Although *Metschnikowia* genus is frequently associated with enhanced aromatic complexity in wine [11,58], β-glucosidase activity was not detected in the two *M. pulcherrima* isolates analysed in the present study. The absence of this activity among the isolates highlights the strongly strain-dependent nature of this trait, as previously reported [47]. There are conflicting reports regarding β-glucosidase activity in *Saccharomyces* yeasts; in many cases, the reported enzyme yield and activity are low or even absent, with significant variability observed among *S. cerevisiae* strains [47]. In this context, a recent study [59] showed that, among ten *S. cerevisiae* strains evaluated, only two were able to hydrolyse 21 out of 93 compounds. The remaining strains exhibited variable hydrolysis patterns, further supporting the strain-dependent differences in glycosidase activity [59].

Additional enzymatic activities evaluated in the present work included those associated with flavour enhancement and haze reduction (protease and pectinase). A high prevalence of pectinase activity was observed in both *S. cerevisiae* and *H. guilliermondii* isolates. Protease activity was more frequently detected among *H. guilliermondii* strains and was less common in *S. cerevisiae*, although some *S. cerevisiae* isolates were positive for this trait. Notably, all yeast strains exhibited at least one enzymatic activity of oenological interest, and a substantial proportion of the isolates displayed two or more hydrolytic activities. The *S. cerevisiae* and the NS isolates examined displayed these activities in a strain-dependent manner as it was previously reported [47,60,61], further supporting their potential contribution to wine sensory modulation.

Regarding additional oenological traits evaluated, ethanol tolerance represented the most evident and expected difference between species. *S. cerevisiae* strains were able to tolerate ethanol concentrations up to 15%, whereas NS isolates generally exhibited lower tolerance levels. High ethanol tolerance is a well-recognised oenological trait that contributes to the predominance of *S. cerevisiae* during grape must fermentation [11]. The progressive decline of NS species throughout fermentation has traditionally been attributed to their limited capacity to withstand increasing ethanol concentrations, with a maximum tolerance of approximately 6% (*v*/*v*) frequently reported [62]. In the present study, ethanol tolerance in the NS group was evaluated up to 10%, and several isolates were able to grow at this concentration, indicating a greater tolerance than previously described. Apiculate yeasts are generally considered to be poorly tolerant to ethanol; however, the *H. uvarum* isolates analysed here demonstrated higher tolerance levels than previously assumed. Furthermore, certain NS species, including *H. guilliermondii* and *Starmerella stellata* (formely *Candida stellata*), have been reported to exhibit greater ethanol tolerance than formerly recognised [52,63]. The findings of the present study are consistent with these observations.

Additionally, given the extensive phenotypic diversity previously reported among isolates of *Hanseniaspora* species [50], the present study confirmed a generally positive response of strains belonging to this genus under the stressful conditions evaluated, including low and high growth temperatures, low pH, and the presence of SO_2_. The only exception was observed in two *H. guilliermondii* isolates, which exhibited reduced tolerance to these conditions. This finding further supports the marked intra-genus variability and the strain-dependent nature of stress response traits within *Hanseniaspora* [30,52].

*Saccharomyces cerevisiae* is the primary fermentative yeast and the microorganism most widely employed in wine, bread, and beer production. Several physiological and metabolic traits position *S. cerevisiae* as the main oenological species. Notably, the Crabtree effect enables this yeast, even under aerobic conditions, to preferentially ferment sugars rather than fully respire them, leading to ethanol and other two-carbon compound production instead of increased biomass formation. Consequently, *S. cerevisiae* rapidly accumulates ethanol, creating inhibitory conditions that limit the growth of competing microorganisms [11]. In this context, the results obtained in the present study are consistent with the expected behaviour of *S. cerevisiae* strains, which exhibited high fermentation rates, short lag phases, efficient sugar consumption, and high ethanol yields. Nevertheless, considerable intraspecific variability was observed, reinforcing the importance of screening these traits during the early stages of wine yeast selection programmes.

In contrast, NS yeasts comprise a broad and metabolically diverse group of species. Several NS species exhibit distinct respiro-fermentative regulatory mechanisms compared with *S. cerevisiae*, allowing them to redirect carbon flux towards metabolites other than ethanol. Under aerobic conditions, particularly during the early stages of winemaking, certain species are able of metabolising sugars predominantly through respiratory pathways (i.e., a negative Crabtree effect) [64,65]. However, this trait is not universally distributed among NS yeasts. Some species, such as *S. stellata* and apiculate yeasts belonging to the genera *Hanseniaspora*/*Kloeckera*, have been described as possessing limited fermentative power. Conversely, other NS species, including *Schizosaccharomyces pombe*, *Saccharomycodes ludwigii*, and *Torulaspora delbrueckii*, have demonstrated considerable fermentative capacity [66]. Studies focusing on *Hanseniaspora* species have highlighted marked strain-dependent variability, with certain *H. guilliermondii* strains exhibiting substantial ethanol production and tolerance [10,60]. These observations are consistent with the findings of the present study, in which the NS group was generally characterised by lower fermentation rates, extended lag phases, reduced ethanol production efficiency, and lower sugar uptake compared with *S. cerevisiae*. Nevertheless, an exception was identified in *H. guilliermondii* strain G213, which displayed a particularly noteworthy fermentative profile.

Additionally, acetic acid production has frequently been associated with certain NS species, particularly apiculate yeasts. However, under the grape must fermentation conditions evaluated in the present study, the NS isolates generally produced lower amounts of acetic acid than *S. cerevisiae* strains. In agreement with these findings, Testa et al. [30] screened a large collection of *Hanseniaspora* strains (193 strains) and reported marked intraspecific variability in oenological traits. Notably, more than 60% of the evaluated *H. guilliermondii* and *H. uvarum* strains produced less than 0.75 g/L of acetic acid, highlighting the strain-dependent nature of this parameter. Several studies have characterised diverse NS species for different oenological applications, leading to their selection as candidates for co-inoculated or sequential starter cultures [67,68,69].

The integration of multiple oenological traits under two contrasting scenarios enabled the identification of six yeast strains best suited to each technological objective. Non-*Saccharomyces* isolates predominated in the low-ethanol scenario, whereas *S. cerevisiae* strains were dominant under conditions favouring high ethanol production. This approach highlights the potential of scenario-based selection strategies to tailor yeast combinations according to specific winemaking goals and desired wine styles. Such findings support the design of mixed or sequential inoculation strategies, in which different yeast species can be combined to exploit their complementary metabolic capabilities. Future studies involving pilot-scale fermentations using *criolla* grape musts and selected yeast combinations will be essential to evaluate their impact on fermentation kinetics as well as on the chemical and sensory profile of the resulting wines.

Finally, the characterisation and selection approach applied in the present study aims to support the development of tailored starter cultures for the production of genuinely Argentine *criolla* wines. It provides a robust foundation for the future development of regional starter cultures and the valorisation of *criolla*-based wines.

## Figures and Tables

**Table 1:** Ranking of yeast isolates according to their overall desirability in two different scenarios.

Species	Isolate	First Scenario ^1^	Species	Isolate	Second Scenario ^2^
*H. guilliermondii*	C27	0.471	*S. cerevisiae*	B45	0.767
*M. pulcherrima*	C12	0.463	*S. cerevisiae*	B42	0.752
*H. guilliermondii*	D21	0.449	*S. cerevisiae*	G47	0.702
*S. cerevisiae*	E420	0.441	*S. cerevisiae*	E420	0.693
*S. cerevisiae*	B45	0.420	*S. cerevisiae*	G48	0.660
*H. guilliermondii*	G217	0.416	*S. cerevisiae*	A320	0.606
*S. cerevisiae*	A320	0.401	*H. guilliermondii*	G213	0.509
*H. guilliermondii*	A36	0.395	*S. cerevisiae*	E39	0.415
*H. guilliermondii*	C24	0.381	*M. pulcherrima*	C11	0.370
*S. cerevisiae*	G48	0.368	*H. guilliermondii*	G217	0.301
*S. cerevisiae*	G47	0.325	*H. guilliermondii*	A36	0.290
*S. cerevisiae*	B42	0.316	*H. guilliermondii*	C27	0.283
*H. uvarum*	C114	0.309	*H. guilliermondii*	C24	0.262
*H. guilliermondii*	G213	0.281	*H. guilliermondii*	D21	0.238
*M. pulcherrima*	C11	0.222	*M. pulcherrima*	C12	0.231
*S. cerevisiae*	E39	0.086	*H. uvarum*	C114	0.196

The first scenario ^1^ prioritised fast growth, a short lag phase, complete sugar consumption, low ethanol and acetic acid production, and high fermentative purity and yield, aiming to favour the formation of metabolites other than ethanol. The second scenario ^2^ prioritised fast growth, a short lag phase, complete sugar consumption, low acetic acid production, and maximum ethanol production, favouring efficient sugar-to-ethanol conversion. Shaded cells indicate the selected yeast strains.

## Data Availability

The raw data supporting the conclusions of this article will be made available by the authors on request.

## Supplementary Materials

The following supporting information can be downloaded at: https://www.mdpi.com/article/10.3390/jof12050322/s1, Table S1: Characterisation of yeast isolates based on 5.8S-ITS region and RFLP patterns and sequence identification using 5.8S-ITS and 26S-D1/D2 domain; Table S2: Contingency table of oenological traits and species distribution of yeast isolates; Table S3: Fermentative and metabolic parameters of isolates obtained in small-scale fermentations; Table S4: Desirability analysis for 2 scenarios. Optimisation goals, and relative importance assigned to fermentation parameters considered for each desirability scenario. Figure S1: Discriminant analysis of yeast isolates based on fermentation parameters.

## References

[1] Uvas Criollas: El Origen de un Vino de Calidad y Con Historia. https://intainforma.inta.gob.ar/uvas-criollas-el-origen-de-un-vino-de-calidad-y-con-historia/.

[2] Prieto J.A. (2021). Vinos y Variedades Patrimoniales: Resumen de las Primeras Jornadas Latinoamericanas.

[3] Aliquó G., Torres R., Lacombe T., Boursiquot J.M., Laucou V., Gualpa J., Fanzone M., Sari S., Pérez Peña J., Prieto J.A. (2017). Identity and parentage of some South American grapevine cultivars present in Argentina. Aust. J. Grape Wine Res..

[4] Informe Anual de Superficie 2024. Instituto Nacional de Vitivinicultura (INV). http://www.inv.gob.ar.

[5] Rui M., Blanc S., Brun F., Massaglia S. (2025). Shifting wine consumption trends (2019–2024): Market dynamics, sustainability, and consumer preferences. Proceedings of the 46th World Congress of Vine and Wine.

[6] Actualización del Plan Estratégico Vitivinícola al 2030. Corporación Vitivinícola Argentina (COVIAR). https://www.coviar.com.ar.

[7] Ribéreau-Gayon P., Dubourdieu D., Donèche B., Lonvaud A. (2021). Handbook of Enology, Volume 1: The Microbiology of Wine and Vinifications.

[8] Pretorius I.S. (2020). Tasting the terroir of wine yeast innovation. FEMS Yeast Res..

[9] Borren E., Tian B. (2020). The important contribution of non-*Saccharomyces* yeasts to the aroma complexity of wine: A review. Foods.

[10] Knight S., Goddard M.R. (2015). Quantifying separation and similarity in a *Saccharomyces cerevisiae* metapopulation. ISME J..

[11] Fazio N.A., Russo N., Foti P., Pino A., Caggia C., Randazzo C.L. (2023). Inside current winemaking challenges: Exploiting the potential of conventional and unconventional yeasts. Microorganisms.

[12] Padilla B., García-Fernández D., González B., Izidoro I., Esteve-Zarzoso B., Beltran G., Mas A. (2016). Yeast biodiversity from DOQ Priorat uninoculated fermentations. Front. Microbiol..

[13] Loira I., Morata A., Bañuelos M.A., Suárez-Lepe J.A. (2020). Isolation, selection, and identification of oenological yeasts. Biotechnological Progress and Beverage Consumption.

[14] Castrillo D., Blanco P. (2023). Characterization of indigenous non-*Saccharomyces* yeast strains with potential use in winemaking. Front. Biosci..

[15] Canonico L., Solomon M., Comitini F., Ciani M., Varela C. (2019). Volatile profile of reduced alcohol wines fermented with selected non-*Saccharomyces* yeasts under different aeration conditions. Food Microbiol..

[16] Maturano Y.P., Mestre M.V., Kuchen B., Toro M.E., Mercado L.A., Vázquez F., Combina M. (2019). Optimization of fermentation-relevant factors: A strategy to reduce ethanol in red wine by sequential culture of native yeasts. Int. J. Food Microbiol..

[17] Minnaar P.P., du Plessis H.W., Jolly N.P., van der Rijst M., du Toit M. (2019). Non-Saccharomyces yeast and lactic acid bacteria in co-inoculated fermentations with two Saccharomyces cerevisiae yeast strains: A strategy to improve the phenolic content of Syrah wine. Food Chem. X.

[18] Castrillo D., Rabuñal E., Neira N., Blanco P. (2019). Oenological potential of non-*Saccharomyces* yeasts to mitigate effects of climate change in winemaking: Impact on aroma and sensory profiles of Treixadura wines. FEMS Yeast Res..

[19] Wei J., Zhang Y., Wang Y., Ju H., Niu C., Song Z., Yuan Y., Yue T. (2020). Assessment of chemical composition and sensorial properties of ciders fermented with different non-*Saccharomyces* yeasts in pure and mixed fermentations. Int. J. Food Microbiol..

[20] Belda I., Ruiz J., Alastruey-Izquierdo A., Navascués E., Marquina D., Santos A. (2016). Unraveling the enzymatic basis of wine “flavorome”: A phylo-functional study of wine-related yeast species. Front. Microbiol..

[21] Escribano-Viana R., González-Arenzana L., Portu J., Garijo P., López-Alfaro I., López R., Santamaría P., Gutiérrez A.R. (2018). Wine aroma evolution throughout alcoholic fermentation sequentially inoculated with non-*Saccharomyces*/*Saccharomyces* yeasts. Food Res. Int..

[22] Binati R.L., Lemos Junior W.J.F., Luzzini G., Slaghenaufi D., Ugliano M., Torriani S. (2020). Contribution of non-Saccharomyces yeasts to wine volatile and sensory diversity: A study on *Lachancea thermotolerans, Metschnikowia spp.* and *Starmerella bacillaris* strains isolated in Italy. Int. J. Food Microbiol..

[23] Aplin J.J., White K.P., Edwards C.G. (2019). Growth and metabolism of non-*Saccharomyces* yeasts isolated from Washington State vineyards in media and high sugar grape musts. Food Microbiol..

[24] Bougreau M., Ascencio K., Bugarel M., Nightingale K., Loneragan G. (2019). Yeast species isolated from Texas High Plains vineyards and dynamics during spontaneous fermentations of Tempranillo grapes. PLoS ONE.

[25] Chen Y., Jiang J., Song Y., Zang X., Wang G., Pei Y., Song Y., Qin Y., Liu Y. (2022). Yeast diversity during spontaneous fermentations and oenological characterisation of indigenous *Saccharomyces cerevisiae* for potential as wine starter cultures. Microorganisms.

[26] Iturritxa E., Hill A.E., Torija M.-J. (2023). Profiling potential brewing yeast from forest and vineyard ecosystems. Int. J. Food Microbiol..

[27] Albertin W., Setati M.E., Miot-Sertier C., Mostert T.T., Colonna-Ceccaldi B., Coulon J., Girard P., Moine V., Pillet M., Salin F. (2016). Hanseniaspora uvarum from winemaking environments show spatial and temporal genetic clustering. Front. Microbiol..

[28] Lombardi S.J., Pannella G., Iorizzo M., Moreno-Arribas M.V., Tremonte P., Succi M., Sorrentino E., Macciola V., Di Renzo M., Coppola R. (2018). Sequential inoculum of *Hanseniaspora guilliermondii* and *Saccharomyces cerevisiae* for winemaking Campanino on an industrial scale. World J. Microbiol. Biotechnol..

[29] Mestre M.V., Maturano Y.P., Gallardo C., Combina M., Mercado L., Toro M.E., Carrau F., Vazquez F., Dellacassa E. (2019). Impact on sensory and aromatic profile of low ethanol Malbec wines fermented by sequential culture of *Hanseniaspora uvarum* and *Saccharomyces cerevisiae* native yeasts. Fermentation.

[30] Testa B., Lombardi S.J., Iorizzo M., Letizia F., Di Martino C., Di Renzo M., Strollo D., Tremonte P., Pannella G., Ianiro M. (2020). Use of strain *Hanseniaspora guilliermondii* BF1 for winemaking process of white grapes *Vitis vinifera* cv. Fiano. Eur. Food Res. Technol..

[31] Bokulich N.A., Collins T.S., Masarweh C., Allen G., Heymann H., Ebeler S.E., Mills D.A. (2016). Associations among wine grape microbiome, metabolome, and fermentation behavior suggest microbial contribution to regional wine characteristics. mBio.

[32] Paolinelli M., Martínez L., Brond V., García R., Sari S., Catania A., Uliarte M., Lerena M.C., Chimeno S.V., García Lampasona S. (2026). Wild microbial communities shaping the spontaneous fermentation of biodynamic Malbec wine. OENO One.

[33] Hoffman C.S., Winston F.A. (1987). A ten minutes preparation from yeast efficiently releases autonomous plasmids for transformation of *Escherichia coli*. Gene.

[34] Esteve-Zarzoso B., Belloch C., Uruburu F., Querol A. (1999). Identification of yeasts by RFLP analysis of the 5.8S rRNA gene and the two ribosomal internal transcribed spacers. Int. J. Syst. Evol. Microbiol..

[35] Granchi L., Bosco M., Messini A., Vincenzini M. (1999). Rapid detection and quantification of yeast species during spontaneous wine fermentation by PCR–RFLP analysis of the rRNA ITS region. J. Appl. Microbiol..

[36] White T.J., Bruns T.D., Lee S.B., Taylor J.W., Innis M.A., Gelfand D.H., Sninsky J.J., White T.J. (1990). Amplification and direct sequencing of fungal ribosomal RNA genes for phylogenetics. PCR Protocols: A Guide to Methods and Applications.

[37] Kurtzman C.P., Fell J.W., Boekhout T. (2011). The Yeasts, a Taxonomic Study.

[38] Fell J.W., Boekhout T., Fonseca A., Scorzetti G., Statzell-Tallman A. (2000). Biodiversity and systematics of basidiomycetous yeasts as determined by large-subunit rDNA D1/D2 domain sequence analysis. Int. J. Syst. Evol. Microbiol..

[39] Massera A., Assof M., Sturm M.E., Sari S., Jofré V., Cordero-Otero R., Combina M. (2012). Selection of indigenous *Saccharomyces cerevisiae* strains to ferment red musts at low temperature. Ann. Microbiol..

[40] Mendes-Ferreira A., Mendes-Faia A., Leão C. (2002). Survey of hydrogen sulphide production by wine yeasts. J. Food Prot..

[41] Caridi A., Cufari A., Ramondino D. (2002). Isolation and clonal pre-selection of enological *Saccharomyces*. J. Gen. Appl. Microbiol..

[42] Hernández L.F., Espinosa J.C., Fernández-González M., Briones A. (2003). β-Glucosidase activity in a *Saccharomyces cerevisiae* wine strain. Int. J. Food Microbiol..

[43] Fernandes-Salomão T.M., Amorim A.C.R., Chaves-Alves V.M., Coelho J.L.C., Silva D.O., de Araújo E.F. (1996). Isolation of pectinase hyperproducing mutants of *Penicillium expansum*. Rev. Microbiol..

[44] Merín M.G., Mendoza L.M., Farías M.E., Morata de Ambrosini V.I. (2011). Isolation and selection of yeasts from wine grape ecosystem secreting cold-active pectinolytic activity. Int. J. Food Microbiol..

[45] Comitini F., Gobbi M., Domizio P., Romani C., Lencioni L., Mannazzu I., Ciani M. (2011). Selected non-*Saccharomyces* wine yeasts in controlled multistarter fermentations with *Saccharomyces cerevisiae*. Food Microbiol..

[46] (2020). InfoStat.

[47] Zhang P., Zhang R., Sirisena S., Gan R., Fang B. (2021). Beta-glucosidase activity of wine yeasts and its impacts on wine volatiles and phenolics: A mini-review. Food Microbiol..

[48] Loureiro V., Malfeito-Ferreira M. (2003). Spoilage yeasts in the wine industry. Int. J. Food Microbiol..

[49] Combina M., Elía A., Mercado L., Catania C., Ganga A., Martinez C. (2005). Dynamics of indigenous yeast populations during spontaneous fermentation of wines from Mendoza, Argentina. Int. J. Food Microbiol..

[50] Combina M., Mercado L., Borgo P., Elía A., Jofré V., Ganga A., Martinez C., Catania C. (2005). Yeasts associated to Malbec grape berries from Mendoza, Argentina. J. Appl. Microbiol..

[51] Clark K., Setati M., Divol B. (2025). Exploring the oenological potential of South African *Saccharomyces cerevisiae* isolates. OENO One.

[52] Onetto C.A., Ward C.M., Varela C., Hale L., Schmidt S.A., Borneman A.R. (2025). Genetic and phenotypic diversity of wine-associated *Hanseniaspora* species. FEMS Yeast Res..

[53] Sánchez M.L., Chimeno S.V., Mercado L.A., Ciklic I.F. (2022). Hybridization and spore dissection of native wine yeasts for improvement of ethanol resistance and osmotolerance. World J. Microbiol. Biotechnol..

[54] Sidari R., Ženišová K., Tobolková B., Belajová E., Cabicarová T., Bučková M., Puškárová A., Planý M., Kuchta T., Pangallo D. (2021). Wine yeasts selection: Laboratory characterization and protocol review. Microorganisms.

[55] Zhang P., Ma W., Meng Y., Zhang Y., Jin G., Fang Z. (2021). Wine phenolic profile altered by yeast: Mechanisms and influences. Compr. Rev. Food Sci. Food Saf..

[56] Liang Z., Fang Z., Pai A., Luo J., Gan R., Gao Y., Lu J., Zhang P. (2020). Glycosidically bound aroma precursors in fruits: A comprehensive review. Crit. Rev. Food Sci. Nutr..

[57] Maicas S., Mateo J.J. (2005). Hydrolysis of terpenyl glycosides in grape juice and other fruit juices: A review. Appl. Microbiol. Biotechnol..

[58] Lee S.-B., Park H.-D. (2020). Isolation and investigation of potential non-*Saccharomyces* yeasts to improve the volatile terpene compounds in Korean Muscat Bailey A wine. Microorganisms.

[59] Piao H., Collins T.S., Henick-Kling T. (2025). Investigation of smoke-taint precursor modification by glycosidase activity in diverse wine yeast and bacterial strains. Front. Microbiol..

[60] Ianieva O., Podgorsky V. (2021). Enological potential of non-*Saccharomyces* yeast strains of enological and brewery origin from Ukrainian Collection of Microorganisms. Mycology.

[61] van Wyk N., Badura J., von Wallbrunn C., Pretorius I.S. (2024). Exploring future applications of the apiculate yeast *Hanseniaspora*. Crit. Rev. Biotechnol..

[62] Fleet G.H. (2008). Wine yeasts for the future. FEMS Yeast Res..

[63] Pina C., Santos C., Couto J.A., Hogg T. (2004). Ethanol tolerance of five non-*Saccharomyces* wine yeasts in comparison with a strain of *Saccharomyces cerevisiae*: Influence of different culture conditions. Food Microbiol..

[64] Quirós M., Rojas V., Gonzalez R., Morales P. (2014). Selection of non-*Saccharomyces* yeast strains for reducing alcohol levels in wine by sugar respiration. Int. J. Food Microbiol..

[65] Ciani M., Morales P., Comitini F., Tronchoni J., Canonico L., Curiel J.A., Oro L., Rodrigues A.J., Gonzalez R. (2016). Non-conventional yeast species for lowering ethanol content of wines. Front. Microbiol..

[66] Querol A., Fleet G.H. (2006). Yeasts in Food and Beverages.

[67] Padilla B., Gil J.V., Manzanares P. (2016). Past and future of non-*Saccharomyces* yeasts: From spoilage microorganisms to biotechnological tools for improving wine aroma complexity. Front. Microbiol..

[68] Mestre Furlani M.V., Maturano Y.P., Combina M., Mercado L.A., Toro M.E., Vazquez F. (2017). Selection of non-*Saccharomyces* yeasts to be used in grape musts with high alcoholic potential: A strategy to obtain wines with reduced ethanol content. FEMS Yeast Res..

[69] Morata A., Arroyo T., Bañuelos M.A., Blanco P., Briones A., Cantoral J.M., Castrillo D., Cordero-Bueso G., Del Fresno J.M., Escott C. (2023). Wine yeast selection in the Iberian Peninsula: *Saccharomyces* and non-*Saccharomyces* as drivers of innovation in Spanish and Portuguese wine industries. Crit. Rev. Food Sci. Nutr..

