# Supplementary material for: Profiling Potential Wine Yeast Starters from Criolla Grape Varieties from Argentina
Source: J Fungi (Basel). 2026 Apr 28;12(5):322. doi: 10.3390/jof12050322 (PMC13208210; doi:10.3390/jof12050322)
Supplement: Supplementary file 1 [file jof-12-00322-s001.zip › jof-4215056-supplementary.pdf]

Table S1. Characterization of yeast isolates based on 5.8S-ITS region and RFLP patterns and sequence identification using 5.8S-ITS and 26S-D1/D2 domain.

Profile	Isolates	Fragment lengths (bp)					5.8S-RFLP identification	Sequencing identification	
		PCR	CfoI/HhaI	HaeIII	HinfI	DdeI	Yeast-ID.org database	5.8S-ITS	26S D1/D2
A	307	850	375, 325, 150	325, 230, 170, 125	375, 365, 110	-	*S. cerevisiae (100%)*	*S. cerevisiae*	-
B	91	775	340, 320, 105	775	350, 200, 150	370, 180, 130, 90	*H. guilliermondii (80%)*	*H. guilliermondii*	*H. guilliermondii*
C	48	750	340, 320, 100	775	350, 200, 180	290, 180, 100, 90	*H. uvarum (80%)*	*H. uvarum*	-
D	13	400	210, 100, 95	290, 120	200, 190	-	*M. pulcherrima (100%)*	*M. pulcherrima*	*M. pulcherrima*
E	9	450	190, 110	450	230, 210	-	*C. stellata (100%)*	*S. bacillaris*	-
F	4	800	320, 220, 150, 120	700	400, 380	-	*T. delbruekii (50%)*	*T. delbruekii*	-
G	4	420	200, 90, 50, 40	280, 70	210, 190	-	*M. pulcherrima (88%)*	*C. californica*	-
H	2	550	280, 250	380, 100	260	-	*Cryptococcus laurenti (100%)*	*C. parapsilosis*	-
I	1	700	300, 280	650	350, 180, 150	-	*Kluyveromyces thermotolerans(75%)*	*C. boidinii*	-
J	4	450	150, 100, 90	300, 160, 150	260, 240, 120, 50	-	*Issatchenkia terricola (88%)*	*Pichia terricola*	-
K	1	700	300, 260, 90	480, 200	400, 300		*Saccharomyces kunashirensis (75%)*	*Ogataea uvarum*	-
L	1	750	270, 180, 150, 90	660, 110	390, 370	460, 230, 80	*H. vinae (100%)*	*H. vinae*	*H. vinae*

*Percentages indicate similarity values obtained from the Yeast-ID.org database. Species names reported correspond to the final identification based on sequencing data. Dashes (–) indicate that the corresponding analysis was not performed for those species or profiles.

Table S2. Contingency table of oenological traits and species distribution of yeast isolates

Glucosidase activity	Ethanol tolerance	Protease activity	Pectinase activity	SO_2 tolerance	Osmotic resistance	Growth at 35 °C	Growth at 15 °C	Growth at pH 3,5	*H. guilliermondii*	*H. uvarum*	*M. pulcherrima*	*S. cerevisiae*	Total
0	1	0	1	1	1	1	1	1	0	0	0	26	26
0	1	1	1	1	1	1	1	1	0	0	2	2	4
1	0	0	0	1	0	0	0	0	1	0	0	0	1
1	0	0	0	1	1	1	1	1	7	0	0	0	7
1	0	0	1	1	1	1	1	1	27	0	0	0	27
1	0	1	0	1	1	1	1	1	1	0	0	0	1
1	0	1	1	1	0	0	0	0	1	0	0	0	1
1	0	1	1	1	1	1	1	1	6	0	0	0	6
1	1	0	0	1	1	1	1	1	6	1	0	0	7
1	1	0	1	1	1	1	1	1	1	1	0	5	7
1	1	1	1	1	1	1	1	1	15	0	0	2	17
Total									65	2	2	35	104

* Binary values indicate the absence (0) or presence (1) of each positive oenological trait. Columns corresponding to yeast species indicate the number of isolates displaying each combination of traits.

Table S3. Fermentative and metabolic parameters of isolates obtained in small-scale fermentations.

Species	Strain	Fermentation rate (μ_{max})	Lag phase (λ)	Ethanol production (% v/v)	AA production (g/L)	Sugar uptake (%)	Fermentative yield	Fermentation efficiency (%)
S. cerevisiae	A320	17.02 ± 0.83^{defg}	0.94 ± 0.15^{cde}	11.90 ± 0.26^{b}	0.86 ± 0.05^{b}	82.26 ± 2.42^{b}	17.48 ± 1.16^{ab}	79.20 ± 1.75^{b}
S. cerevisiae	B42	20.56 ± 0.83^{g}	−0.30 ± 0.15^{ab}	13.63 ± 0.26^{cd}	0.84 ± 0.05^{b}	86.07 ± 2.42^{b}	16.01 ± 1.16^{a}	90.66 ± 1.75^{cd}
S. cerevisiae	B45	19.32 ± 0.83^{fg}	−0.51 ± 0.15^{a}	13.15 ± 0.26^{bcd}	0.78 ± 0.05^{ab}	88.98 ± 2.42^{b}	17.12 ± 1.16^{ab}	87.49 ± 1.75^{bcd}
S. cerevisiae	E39	16.16 ± 0.83^{cdef}	0.62 ± 0.15^{cde}	14.29 ± 0.26^{d}	1.14 ± 0.05^{c}	87.43 ± 2.42^{b}	15.48 ± 1.16^{a}	95.05 ± 1.75^{d}
S. cerevisiae	E420	16.88 ± 0.83^{defg}	0.96 ± 0.15^{cde}	12.72 ± 0.26^{bc}	0.76 ± 0.05^{ab}	94.29 ± 2.42^{b}	18.76 ± 1.16^{abcd}	84.61 ± 1.75^{bc}
S. cerevisiae	G47	17.19 ± 0.83^{efg}	−0.30 ± 0.15^{ab}	13.46 ± 0.26^{cd}	0.88 ± 0.05^{b}	87.56 ± 2.42^{b}	16.48 ± 1.16^{a}	89.58 ± 1.75^{cd}
S. cerevisiae	G48	16.91 ± 0.83^{defg}	0.33 ± 0.15^{bcd}	13.06 ± 0.26^{bcd}	0.89 ± 0.05^{b}	89.20 ± 2.42^{b}	17.31 ± 1.16^{ab}	86.87 ± 1.75^{bcd}
H. guilliermondii	A36	14.51 ± 0.83^{bcde}	0.26 ± 0.15^{abc}	6.14 ± 0.26^{a}	0.76 ± 0.05^{ab}	45.69 ± 2.42^{a}	18.85 ± 1.16^{abcd}	40.83 ± 1.75^{a}
M. pulcherrima	C11	11.80 ± 0.83^{ab}	2.39 ± 0.15^{f}	11.81 ± 0.26^{b}	0.71 ± 0.05^{ab}	82.38 ± 2.42^{b}	17.65 ± 1.16^{abc}	78.55 ± 1.75^{b}
H. uvarum	C114	12.75 ± 0.83^{bcd}	0.97 ± 0.15^{cde}	5.75 ± 0.26^{a}	0.86 ± 0.05^{b}	44.75 ± 2.42^{a}	19.75 ± 1.16^{abcd}	38.23 ± 1.75^{a}
M. pulcherrima	C12	12.18 ± 0.83^{abc}	0.71 ± 0.15^{cde}	5.04 ± 0.26^{a}	0.55 ± 0.05^{a}	46.59 ± 2.42^{a}	23.15 ± 1.16^{bcd}	33.53 ± 1.75^{a}
H. guilliermondii	C24	12.73 ± 0.83^{bcd}	1.26 ± 0.15^{e}	6.26 ± 0.26^{a}	0.83 ± 0.05^{b}	52.34 ± 2.42^{a}	21.15 ± 1.16^{abcd}	41.67 ± 1.75^{a}
H. guilliermondii	C27	14.14 ± 0.83^{bcde}	1.00 ± 0.15^{cde}	5.94 ± 0.26^{a}	0.73 ± 0.05^{ab}	56.09 ± 2.42^{a}	24.20 ± 1.16^{d}	39.52 ± 1.75^{a}
H. guilliermondii	D21	13.45 ± 0.83^{bcde}	1.06 ± 0.15^{de}	5.39 ± 0.26^{a}	0.68 ± 0.05^{ab}	50.31 ± 2.42^{a}	23.62 ± 1.16^{cd}	35.86 ± 1.75^{a}
H. guilliermondii	G213	8.34 ± 0.83^{a}	1.33 ± 0.15^{e}	12.81 ± 0.26^{bc}	0.80 ± 0.05^{b}	88.32 ± 2.42^{b}	17.45 ± 1.16^{ab}	85.25 ± 1.75^{bc}
H. guilliermondii	G217	13.58 ± 0.83^{bcde}	0.97 ± 0.15^{cde}	6.08 ± 0.26^{a}	0.68 ± 0.05^{ab}	48.24 ± 2.42^{a}	20.18 ± 1.16^{abcd}	40.47 ± 1.75^{a}

*Values are expressed as mean ± standard error (n = 3). Different letters indicate statistical differences among strains according to Tukey's HSD test ($p < 0.05$).

Table S4. Desirability analysis for 2 scenarios. Optimization goals, and relative importance assigned to fermentation parameters considered for each desirability scenario.

Scenario	Variable	Goal	Lower limit	Upper limit	Importance
Low ethanol/low VA	Fermentation rate (μ_{max})	Maximize	7.65	21.34	3
	Lag phase (λ)	Minimize	–1.06	2.40	3
	Ethanol	Minimize	4.40	14.36	5
	Acetic acid	Minimize	0.43	1.16	5
	Sugar uptake	Maximize	32.85	94.70	5
	Sugar/ethanol	Maximize	15.34	29.67	3
	Fermentative purity	Maximize	6.32	24.22	2
High ethanol/low VA	Fermentation rate (μ_{max})	Maximize	7.65	21.34	3
	Lag phase (λ)	Minimize	–1.06	2.40	3
	Ethanol	Maximize	4.40	14.36	5
	Acetic acid	Minimize	0.43	1.16	5
	Sugar uptake	Maximize	32.85	94.70	5
	Sugar/ethanol	Minimize	15.34	29.67	3
	Fermentative efficiency	Maximize	29.27	95.54	3
	Fermentative purity	Maximize	6.32	24.22	4

VA: volatile acitidy

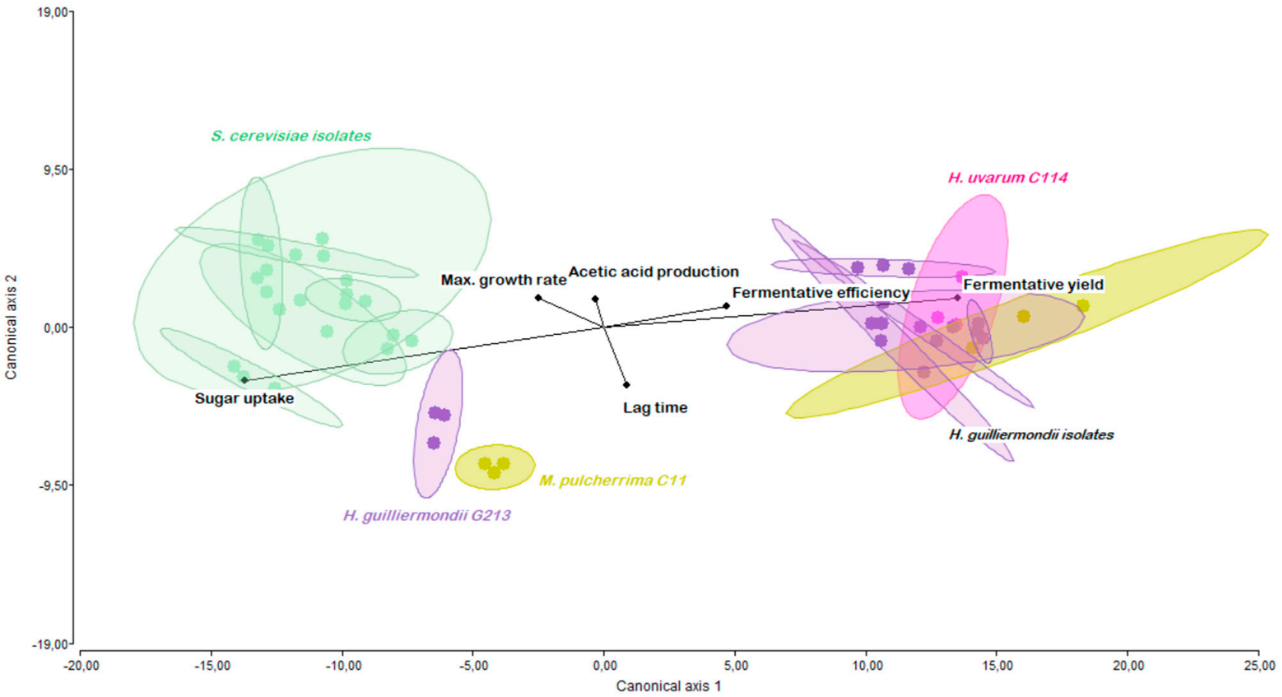

Figure S1. Discriminant analysis of yeast isolates based on fermentation parameters. Confidence ellipses illustrate the grouping of *S. cerevisiae* isolates and selected non-*Saccharomyces* strains (*H. uvarum* C114, *H. guilliermondii* isolates, and *M. pulcherrima* C11). Vectors indicate the contribution of the measured variables to sample discrimination, including sugar uptake, lag time, maximum growth rate, acetic acid production, fermentative efficiency, and fermentative yield.
